# Beneficial Effects of a Short Course of Physical Prehabilitation on Neurophysiological Functioning and Neurovascular Biomarkers in Patients Undergoing Coronary Artery Bypass Grafting

**DOI:** 10.3389/fnagi.2021.699259

**Published:** 2021-12-10

**Authors:** Olga A. Trubnikova, Irina V. Tarasova, Evgeniy G. Moskin, Darya S. Kupriyanova, Yuliya A. Argunova, Svetlana A. Pomeshkina, Olga V. Gruzdeva, Olga L. Barbarash

**Affiliations:** Research Institute for Complex Issues of Cardiovascular Diseases, Kemerovo, Russia

**Keywords:** physical prehabilitation, postoperative cognitive dysfunction, brain electrical activity, S100β, BDNF, coronary artery bypass grafting

## Abstract

This study aimed to evaluate the effects of a short course of physical prehabilitation on neurophysiological functioning and markers of the neurovascular unit in patients undergoing coronary artery bypass grafting (CABG). We performed a prospective randomized study involving 97 male CABG patients aged 45–70 years, 47 of whom underwent a 5–7-day preoperative course of aerobic physical training (PhT). Both groups of patients were comparable with respect to baseline clinical and anamnestic characteristics. An extended neuropsychological and electroencephalographic (EEG) study was performed before surgery and at 7–10 days after CABG. Markers of the neurovascular unit [S100β, neuron-specific enolase (NSE), and brain-derived neurotrophic factor (BDNF)] were examined as metabolic correlations of early postoperative cognitive dysfunction (POCD) at three time points: before surgery, within the first 24 h after surgery, and 7–10 days after CABG. POCD developed in 58% of patients who underwent preoperative PhT, and in 79.5% of patients who did not undergo training, 7–10 days after CABG. Patients without prehabilitation demonstrated a higher percentage of theta1 power increase in the relative change values as compared to the PhT patients (*p* = 0.015). The short preoperative course of PhT was associated with low plasma S100β concentration, but high BDNF levels in the postoperative period. Patients who underwent a short preoperative course of PhT had better cognitive and electrical cortical activity indicators. Markers of the neurovascular unit indicated lower perioperative brain injury after CABG in those who underwent training. A short course of PhT before CABG can decrease the brain’s susceptibility to ischemia and reduce the severity of cognitive impairments in cardiac surgery patients. Electrical brain activity indicators and neurovascular markers, such as S100β and BDNF, can be informative for the effectiveness of cardiac rehabilitation programs.

## Introduction

There is currently a growing interest in research on the ability of physical rehabilitation to resist negative changes in the physical and mental health of patients with cardiovascular diseases, especially after cardiac surgery. It has been established that physical training (PhT) improves not only general physical well-being, but also contributes to positive changes in the lipid profile, hemorheology, and hemodynamics, resulting in decreased body mass and resting heart rate (Argunova et al., 2017). PhT has been shown to help recover the optimal level of physical activity and accelerate patients’ adaptation to different loads in the early postoperative period of coronary artery bypass grafting (CABG) (Argunova et al., 2017, 2018; Doyle et al., 2019). Recent studies focusing on the older population have found that increased physical activity can reduce cognitive decline and provide protection against dementia by acting as a neuroprotective mechanism in normal aging (Voss et al., 2013; Joubert and Chainay, 2018). Several authors have shown that a high level of physical activity is associated with a lower risk of dementia (Aarsland et al., 2010; Lauenroth et al., 2016; Zhou et al., 2020) and lower beta-amyloid accumulation in brain structures (Okonkwo et al., 2014; Merrill et al., 2016). However, the effects of aerobic PhT on cognitive function in patients undergoing cardiac surgery have rarely been studied. For example, a recent study found that a 3-week course of daily PhT after cardiac surgery contributed to the reduction of postoperative cognitive dysfunction (POCD) incidence in patients with coronary artery disease (Argunova et al., 2016). To date, no studies have examined the possible effects of aerobic physical prehabilitation on cognitive function in the postoperative period of cardiovascular surgery. Considering the high medical and social importance of postoperative cognitive disorders in cardiac surgery patients, the intensive search for new ways of preventing this complication continues.

The assessment of the efficacy of prevention methods should include the application of diagnostic tools to identify the factors for recovery progress, as well as a baseline assessment. Quantitative analysis of electroencephalographic (EEG) indices is potentially one of the best candidates among the potential biomarkers of cognitive disorders, because EEG research is a relatively inexpensive, noninvasive, and safe procedure. It has been demonstrated that baseline EEG indices are important for the early diagnosis of Alzheimer’s disease and vascular cognitive impairment (Mazzon et al., 2018; Musaeus et al., 2018; Tarasova et al., 2018).

Physical exercise is closely related to cognitive function through a cascade of cellular and molecular processes that promote angiogenesis, neurogenesis, and synaptogenesis, thus enhancing learning, memory, and brain plasticity (De la Rosa et al., 2019). Peripheral blood biomarkers of brain damage convey information on various pathological states (Ehrenreich et al., 2011; Bustamante et al., 2017; Zetterberg and Burnham, 2019; Onatsu et al., 2020). Several indicators have already been identified, and markers that can reflect the functioning of the neurovascular unit are currently under active study. Among these indicators is S-100 calcium-binding protein B (S100β). This protein is mainly expressed in astrocytes, which allows it to be seen as an acute ischemia marker and is related to the severity of brain damage (He et al., 2021). An increase in serum S100β levels is correlated with neurological deficits, infarct size, and significant brain edema during acute stroke (Onatsu et al., 2020). NSE is another indicator of neurovascular function that is increased in acute ischemic brain damage, which is the worst neurological outcome in stroke (Ehrenreich et al., 2011). NSE is also considered as a marker for postoperative cerebral dysfunction in patients undergoing coronary and carotid artery revascularization (Capoccia et al., 2010; Zheng et al., 2015). A meta-analysis by Zheng et al. (2015) demonstrated that plasma NSE increased within the first 24 h after cardiac surgery, but its predictive role in cerebrovascular outcomes requires further research.

The brain-derived neurotrophic factor (BDNF) has received special attention in the study of restorative mechanisms of cognitive function. Many studies have shown the critical role of BDNF in regulating plastic changes in the adult brain as a key component of the cellular mechanisms underlying the formation and maintenance of memory that promotes synaptic consolidation (Leal et al., 2014; De la Rosa et al., 2019; Wong et al., 2021). Up to 75% of the peripheral blood BDNF is from a brain source, allowing it to be considered an indicator of cerebral BDNF (Pareja-Galeano et al., 2015; De la Rosa et al., 2019). Age-related cognitive decline may be associated with decreased expression in areas of the brain affected by aging, as well as circulating concentrations of BDNF (Shimada et al., 2014).

Some types of training are expected to increase BDNF expression (Szuhany et al., 2015; Tsai et al., 2016; De la Rosa et al., 2019). A meta-review by Szuhany et al. (2015) reported that BDNF levels increased after physical exercises. It can be assumed that preoperative PhT might also be accompanied by an increase in BDNF, and this plastic effect on brain tissue might reduce cognitive deficits in the early postoperative period of cardiac surgery.

Thus, study of the neurophysiological mechanisms underlying cognitive deficits associated with cardiac interventions as well as the search for new diagnostic tools reflecting the effectiveness of recovery procedures is now relevant. The aim of this study was to evaluate the effects of a short course of physical prehabilitation (PhT) on cognitive function, EEG, and markers of the neurovascular unit in patients undergoing CABG.

## Materials and Methods

### Patients

**FIGURE 1:**
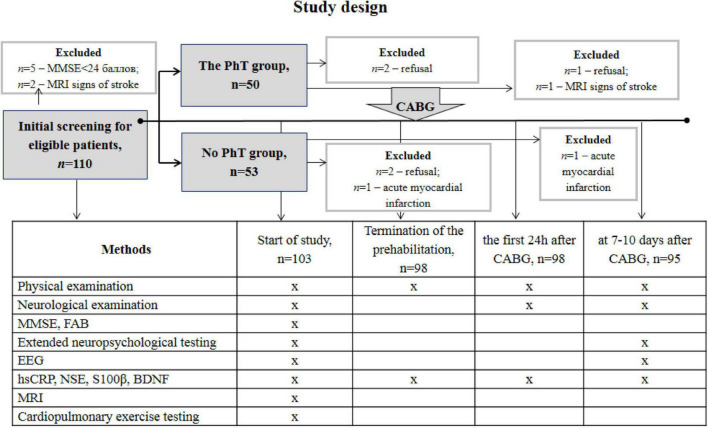
Overview of the study design. PhT, physical training; CON, control.

This prospective randomized study included 103 patients with coronary artery disease aged 45–70 years who were admitted for planned CABG at the Federal State Budgetary Scientific Institution of Research Institute of Complex Issues of Cardiovascular Diseases. The study complied with the Good Clinical Practice standards and the principles of the Declaration of Helsinki. The study protocol was approved by the institutional Ethics Committee (12/20170412). Prior to inclusion in the study, all participants provided written informed consent and underwent neuropsychological screening using the Mini-Mental State Examination (MMSE) scale, assessment of trait and state anxiety according to the Spielberger-Khanin questionnaire, and depression screening according to the Beck Depression Inventory (BDI-II). Patients with depression (BDI-II score ≥ 8) and dementia (MMSE score ≤ 24) were not included in the study. Women were not included in this study to eliminate the effect of sex differences on clinical, demographic, and psychophysiological indicators. The exclusion criteria were as follows: life-threatening rhythm disturbances, chronic heart failure (NYHA functional class III and higher), carotid artery stenosis ≥ 50%, severe comorbid diseases (chronic obstructive pulmonary disease, malignant pathology), drug addiction, stroke, and other brain injuries. Patients who were physically unable to perform aerobic PhT were not included in the study. Envelope-based randomization was used to divide the patients into two groups: the PhT group (*n* = 50) and the group undergoing basic preoperative preparation (*n* = 53). A research team member not involved with the study conducted this procedure. Figure 1 shows the number and distribution of participants included in this study.

All the patients underwent general medical, neurological, and instrumental examinations. The examiners were unaware of the participating in the study of the patients. The patient groups did not differ in their baseline clinical and demographic characteristics (Table 1).

**TABLE 1:** Clinical and demographic characteristics of coronary artery bypass grafting (CABG) patients who did and did not undergo a course of physical prehabilitation.

Variable	Patients with physical prehabilitation	Patients without physical prehabilitation	*p*
	*n* = 50	*n* = 53	
Age, years, Me (Q25; Q75)	59.0 (52; 65)	58.0 (53; 63)	0.29
Body mass index, kg/m2, Me (Q25; Q75)	27.7 (25; 31)	27.3 (25; 30.6)	0.45
Education, *n* (%):			0.98
High-level	18 (38)	20 (40)	
Mid-level	29 (62)	30 (60)	
Smoking, *n* (%)	15 (32)	17 (34)	0.99
Duration of CAD history, years, Me (Q25; Q75)	1.0 (0.5; 5.0)	1.0 (0.5; 7.0)	0.86
FC of angina, *n* (%):			0.96
0–I	6 (13)	6 (12)	
II	41 (87)	44 (88)	
FC NYHA, *n* (%)			
0–I	0 (0)	4 (8)	0.15
II	47 (100)	46 (92)	
History of myocardial infarction, *n* (%)	39 (83)	40 (80)	0.75
Left ventricular EF (%), Me (Q25; Q75)	63 (58; 65)	61 (54; 64)	0.24
History of hypertension, *n* (%)	45 (96)	44 (88)	0.22
Duration of hypertension history, years	4.5 (2.0; 10.0)	3.0 (1.0; 8.0)	0.14
CA stenosis < 50%, *n* (%)	21 (45)	22 (44)	0.94
Type II DM, *n* (%)	10 (21)	13 (26)	0.74
Cholesterol, mmol/l, Me (Q25; Q75)	4.0 (3.3; 5.2)	4.3(3.3; 5.0)	0.85
MMSE, score, Me (Q25; Q75)	28 (27; 29)	28 (27; 29)	0.8
Beck, score, Me (Q25; Q75)	3 (1; 5)	3 (1; 5)	0.99

*CABG, coronary artery bypass grafting; CAD, coronary artery disease; FC, functional class; NYHA, heart failure by the New York Heart Association; EF, ejection fraction; CA, carotid artery; DM, diabetes mellitus; and MMSE, Mini-Mental State Examination.*

Elective CABG was performed in both groups under normothermic non-pulsatile cardiopulmonary bypass (CPB). Standard protocols for combined endotracheal anesthesia and perfusion were used. Intraoperative online monitoring of cerebral cortex oxygenation (rSO_2_) (INVOS-3100, Somanetics, Troy, MI, United States) was performed. The number of grafts (2.5 ± 0.7 vs. 2.5 ± 0.6, *p* = 0.96), mean CPB time (83.5 ± 24.9 vs. 82.6 ± 16.9, *p* = 0.77), and aorta cross-clamping time (53.9 ± 16.8 vs. 52.4 ± 12.0, *p* = 0.66) were similar in both groups.

### Physical Prehabilitation Procedure

The rehabilitation specialist examined the included patients. The training parameters were calculated individually based on cardiopulmonary exercise test data (Cardiovit AT-104 PC Ergo-Spiro, Schiller, Baar, Switzerland). Cardiopulmonary exercise testing (CPET) was performed in the morning, 1–2 h after breakfast. The patient dressed comfortable, loose-fitting clothing and flat shoes performed mild exercise on an upright bicycle. Twelve-channel electrocardiogram (ECG), arterial pressure, ventilation and respiratory gas parameters during exercise was evaluated by the functional diagnostician (Table 2). According to the ACC/AHA Update of Practice Guidelines for Exercise Testing absolute contraindications of CPET are: (1) acute myocardial infarction (3–5 days); (2) unstable angina; (3) uncontrolled arrhythmias causing symptoms or haemodynamic compromise; (4) syncope; (5) active endocarditis, myocarditis or pericarditis; (6) symptomatic severe aortic stenosis; (7) uncontrolled heart failure; (8) acute pulmonary embolus or pulmonary infarction; (9) thrombosis of lower extremities; (10) suspected dissecting aneurysm; and (11) uncontrolled asthma, pulmonary oedema, respiratory failure. Relative contraindications of CPET: (1) left main coronary stenosis or its equivalent; (2) moderate stenotic valvular heart disease; (3) severe untreated arterial hypertension at rest or haemodynamic compromise (>200 mm Hg systolic, >120 mm Hg diastolic); (4) tachyarrhythmias or bradyarrhythmias; (5) high-degree atrioventricular block; 6) hypertrophic cardiomyopathy; (7) significant pulmonary hypertension; and (8) orthopedic impairment that compromises exercise performance.

**TABLE 2:** Cardiopulmonary exercise testing (CPET) indicators in patients who did and did not undergo a course of physical prehabilitation.

Variable, Me (Q25; Q75)	Patients with physical prehabilitation	Patients without physical prehabilitation	*p*
	*n* = 50	*n* = 53	
PV_*O2*_, ml/min/kg	15.3 [14.0; 16.4]	16.8 [12.0; 21.0]	0.27
Maximum heart rate, beats/min	105.0 [96.5; 117.0]	109.5 [97.0; 118.5]	0.21
Ventilatory anaerobic threshold, ml/min/kg	12.0 [12.0; 14.0]	7.55 [5.7; 9.4]	0.06
Exercise tolerance, W	75.0 [75.0; 100.0]	87.5 [75.0; 100.0]	0.09

*PV_O2_, peak oxygen uptake.*

A step-by-step protocol was used. The load was increased gradually every 3 min, with build-up workloads of 25 W/m^2^ until the sub-maximum heart rate or the test termination criteria was reached. Indications for terminating CPET are: (1) symptoms at maximal exercise included muscle fatigue, exhaustion, extreme dyspnoea, and light-headedness; (2) tachyarrhythmias; (3) fall in systolic pressure > 20 mm Hg from the highest value during the test; (4) chest pain suggestive of ischaemia or ischaemic ECG changes; (5) second- or third-degree heart block; 6) signs of respiratory failure; (7) hypertension (>250 mm Hg systolic; >120 mm Hg diastolic); and (8) severe desaturation: Spo2 ≤80% when accompanied by symptoms and signs of severe hypoxaemia.

The peak oxygen consumption (VO_2_ peak) was determined over the last 30 s of peak load and used to select the training load. The anaerobic threshold was determined using the Slope method, a linear regression method for varying the angle of inclination of the VCO_2_/VO_2_ curve. The load accounted for 80% of the maximum oxygen consumption. The parameters of treadmill test were determined considering the following formula: target VO_2_ = 0.1 × (rate) + 1.8 × (rate) × (tilt angle) + 3.5, where target VO_2_ is 80% of VO_2_ peak, rate is measured in m/min, and tilt angle is measured in % (Argunova et al., 2017).

A course of aerobic PhT on a treadmill was implemented once a day for a period of 5–7 days (whilst monitoring heart rate, blood pressure, and electrocardiography data) in the main group, prior to on-pump CABG. The course was conducted on the background of standard medical therapy, including angiotensin-converting enzyme inhibitors/angiotensin II receptor antagonists, beta-blockers, statins, and antiplatelet agents as well as therapeutic and respiratory gymnastics. The daily training session lasted 40 min. The session was performed in the morning, and included a 5-min warm-up and a 5-min cool-down period (walking at a speed of 2.5 km/h in both cases) and a 30-min training phase. The duration of the training phase could be reduced at the patient’s request. The modified Borg scale was used to assess the load level (the optimal load level was 12–15 points).

### Neurophysiological Assessment

The cognitive status assessment included the following typical screening scales: MMSE and FAB in validated Russian-language modified versions. All patients were also examined using extended neuropsychological testing (the assessment of psychomotor and executive function, attention, and short-term memory from the neuropsychological test battery of the psychophysiological complex software “Status PF” (Trubnikova et al., 2014). The detailed description of the neuropsychological tests is shown in Table 3. Multi-channel computed electroencephalography (EEG) was recorded according to methods previously reported (Tarasova et al., 2018).

**TABLE 3:** Cognitive test battery for assessing cognitive function in CABG patients.

Cognitive tests and indicators	Description of the procedure	Reference value
Mini-mental state examination (MMSE)	30-point questionnaire that is used to screen for cognitive impairment and dementia.	≤28 до 30 scores – no cognitive impairment; ≤24 до 27 scores – mild cognitive impairment; ≤20 до 23 scores – mild dementia; ≤11 до 19 scores – moderate dementia; ≤0 до 10 scores – severe dementia
Frontal assessment battery (FAB)	18-point questionnaire that is used to screen for dementia with predominant damage of frontal lobes, evaluate conceptualization, speech fluency, dynamic praxis, reaction of choice, grasping reflexes.	≤16 до 18 scores – no cognitive impairment; ≤12 до 15 scores – mild frontal dysfunction; ≤11 scores – frontal dementia
Complex visual-motor reaction	Reaction latencies of the right and left hands to stimuli (different colors of the rectangles) when the subject should choose one of the three presented signals (the number of signals in the test is 30).	
Reaction time, ms		250.0 ± 15.00
Errors, *n*		0
Level of functional mobility of nervous processes responses to “feedback”	The previous test is conducted in the feedback mode. The duration of the exposure to the test signal (see above) is changed automatically; the exposure of the next signal is shortened by 20 ms with each correct answer and extended by 20 ms, if the answer is wrong (the number of signals in the test is 120).	
Reaction time, ms		280.0 ± 10.3
Errors, *n*		21.0 ± 2.0
Missed signals, *n*		83.0 ± 1.6
Performance of the brain responses to “feedback”	The previous test is conducted in the feedback mode for a fixed period of time (5 min). It is necessary to process the maximum number of signals presented with a given exposure.	
Reaction time, ms		347.0 ± 7.4
Errors, *n*		38.5 ± 7.5
Missed signals, *n*		248.0 ± 4.0
The Bourdon’s test	The subject is provided with the alphabetic version of the Bourdon’s test to highlight certain letters for the lead time of 4 min.	
Processed symbols per 1th min, *n*		
		140.0 ± 20.0
Processed symbols per 4th min, *n*		120.0 ± 20.0
10 words memorizing test, *n*	To remember as many of 10 words presented one after another as possible	6.0 ± 1.0
10 numbers memorizing test, *n*	To remember as many of 10 numbers presented one after another as possible	5.0 ± 1.0
10 nonsense syllable memorizing test, *n*	To remember as many of 10 nonsense syllables presented one after another as possible	3.0 ± 1.0

The PhT group was tested prior to the start of the program, and at 7–10 days after CABG and the control group was tested 5–7 days before surgery and 7–10 days after CABG. Postoperative changes in cognitive indicators were assessed individually for each patient. The percentage of change in indicators was calculated using the formula: [(baseline value – postoperative value) / baseline value] × 100%. A 20% decline in postoperative parameters compared to preoperative parameters in 20% of the test battery indicates POCD (Trubnikova et al., 2014). The original method was used to obtain the integral index of cognitive status (CSI) of patients in the pre- and postoperative CABG periods. CSI was calculated by determining the average distance from the patient’s values to the reference values using the following formula:

$$C\,S\,I=1-\frac{\sqrt{{\left(1-Y\,1\right)}^{2}\,{\left(1-Y\,2\right)}^{2}\,{\left(1-Y\,3\right)}^{2}\,{\left(1-Y\,4\right)}^{2}\,{\left(1-Y\,5\right)}^{2}}}{5},$$

where Y is the recoding value of the cognitive indicator, Y1 is the mean value of the reaction time in psychomotor and executive function tests, Y2 is the mean value of the errors in psychomotor and executive function tests, Y3 is the mean value of the missing signals in psychomotor and executive function tests, Y4 is the mean value of the short-term memory indicator, and Y5 is the mean value of the attention indicator.

### Electroencephalographic Recording

High-resolution monopolar EEG in relaxed wakefulness with eyes closed was recorded. All recordings were made in the first half of the day in light- and sound-proof rooms. Scan 4.5 software and Neuvo SynAmps2 System amplifier (Compumedics, Charlotte, NC, United States) were used [for further details, please refer to Tarasova et al. (2018)]. The average power for each analyzed frequency band [theta1 (4–6 Hz), theta2 (6–8 Hz), alpha1 (8–10 Hz), alpha2 (10–13 Hz), beta1 (13–20 Hz), and beta2 (20–30 Hz)] was calculated.

### Laboratory Data

Whole blood samples were collected from each patient by venipuncture after a 12-h fasting period. To obtain serum, whole blood samples were allowed to coagulate at room temperature for 30 min and then centrifuged at room temperature for 15 min at 1,000 × *g*. The collected serum was stored in polypropylene tubes at −70°C until assayed. The following serum parameters were measured: S100β, NSE, and BDNF. Measurements were made just before the start and after termination of the prehabilitation training course, within the first 24 h after surgery, and at 7–10 days after CABG. Serum concentrations of S100β, NSE, and BDNF were quantitatively determined by using a commercially available enzyme-linked immunosorbent assay ELISA (Bender MedSystems GmbH, Vienna, Austria) [coefficient of variation (CV), 7.03–8.99%].

### Statistical Analysis

All data were analyzed using STATISTICA 10.0 (StatSoft, Tulsa, OK, United States). The normality of the distribution of clinical and demographic parameters was tested using the Kolmogorov-Smirnov test. Most of the clinical indicators were not normally distributed and were analyzed using the Wilcoxon and Mann-Whitney tests. To normalize cognitive status indicators, the integral index of cognitive status (CSI) was calculated and analyzed using a *t*-test for paired and unpaired samples. EEG data were normalized using the logarithm transformation and by calculating the percentage of relative changes in the EEG power after CABG using the formula: [(baseline value – the postoperative value)/baseline value] × 100%. The negative values of the indicator showed an increase, and the positive values showed a decrease in the EEG rhythm power after the surgery compared to baseline. Further analysis of the EEG data was carried out using a single-factor ANOVA. POCD incidence was estimated using relative risk [odds ratio (OR)]. To analyze the factors affecting POCD (dependent variable), a binary logistic regression analysis was used. Age, education, use of PhT, percentage of relative theta1 power changes, S100β, NSE, and BDNF were independent variables identified during the preliminary analysis. The model accuracy was checked by estimating the specificity and sensitivity values. ROC analysis was performed to improve the predictive value of the model.

## Results

### Neuropsychological Results

In our cohort, POCD occurred in 27 patients (58%) in the PhT group and 39 (79.5%) patients who did not undergo physical prehabilitation (OR = 2.74, 95 % CI = 1.11–6.81, *p* = 0.029).

**FIGURE 2:**
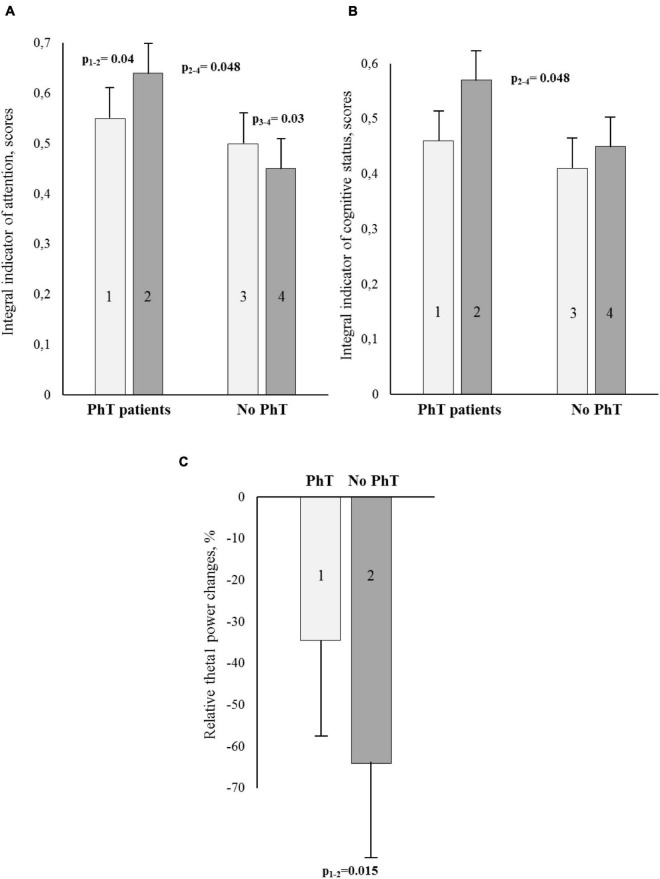
Cognitive and electroencephalographic (EEG) indicators [**A** – attention; **B** – integral indicator of cognitive status; **C** – relative theta1 power changes (%)] for the PhT (physical training) group and the group who did not undergo PhT before and after coronary artery bypass grafting (CABG). **(A,B)** – Light bars indicate preoperative values, dark bars – postoperative ones. Vertical bars denote standard errors (SE).

The psychomotor and executive function, attention, and short-term memory were analyzed, and significant between-group differences were detected for the integral indicator of attention. At 7–10 days after CABG, the attention scores were higher in the PhT group than in the group without prehabilitation (*p* = 0.048) (Figure 2A). In addition, the patients who underwent physical prehabilitation had better attention indicators at 7–10 days after CABG compared to preoperative values (*p* = 0.04), while the patients without who did not undergo physical prehabilitation had worse indicators (*p* = 0.03).

The integral index of psychomotor and executive function in both groups increased at 7–10 days after CABG (*p* = 0.0008 and *p* = 0.0001, respectively). There were no significant differences between the groups.

The improvement of the integral indicator of the cognitive status at 7–10 days after surgery compared to preoperative values was observed only in the PhT group (*p* = 0.06), and statistically significant between-group differences were also observed (*p* = 0.048) (Figure 2B).

### Electroencephalographic Results

One-way ANOVA using the factor GROUP was carried out separately for all studied EEG ranges. The factor was significant for the theta1 range: *F*_1.95_ = 6.29, *p* = 0.015. The patients who did not undergo preoperative PhT demonstrated a higher percentage of theta1 power increase in the relative change values as compared to the patients who did undergo a physical rehabilitation course (see Figure 2C).

### Neurovascular Unit Measurements

**FIGURE 3:**
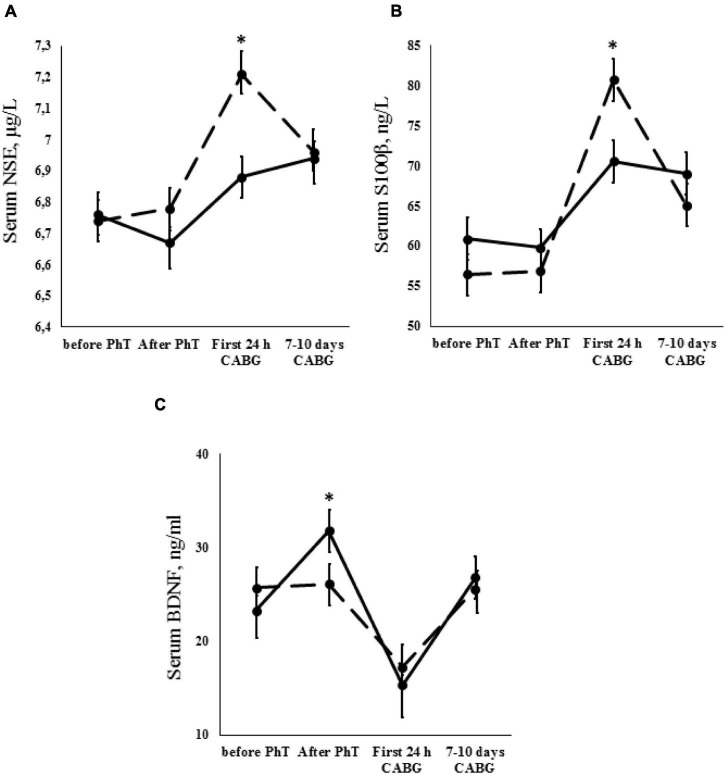
Dynamics of neurovascular unit indicators [**A** – neuron-specific enolase (NSE); **B** – S100β protein; **C** – brain-derived neurotrophic factor (BDNF)] for the PhT group (continuous line) and the group who did not undergo PhT (broken line). Vertical bars denote standard error (SE). Asterisks (*) indicate statistically significant differences (*p* < 0.05) between two groups.

There were no statistically significant differences in the mean NSE concentrations for the PhT group throughout the perioperative period (Figure 3A). However, serum NSE increased in the first 24 h after CABG in patients who did not undergo physical prehabilitation, and subsequently decreased at 7–10 days after surgery (*p* ≤ 0.01). No significant between-group differences were observed in the analysis of preoperative NSE concentrations. During the first 24 h after CABG, NSE concentration was significantly higher in patients who did not undergo physical prehabilitation compared to PhT patients (*p* ≤ 0.01).

Analysis of the S100β protein serum concentrations revealed significant results in both groups. An increase in S100β protein concentrations was found during the first 24 h after surgery (*p* ≤ 0.01) and subsequently decreased at 7–10 days after surgery (*p* ≤ 0.01). However, S100β protein serum concentrations were higher in patients who did not undergo physical prehabilitation compared to the PhT group during the first 24 h after CABG (*p* ≤ 0.01) (Figure 3B).

Analysis of the serum BDNF concentrations in patients who did and did not undergo PhT was carried out. Serum BDNF was increased in PhT patients after termination of the short course of physical prehabilitation (*p* ≤ 0.01). These levels then dropped during the first 24 h after CABG and increased slightly at 7–10 days after surgery (*p* ≤ 0.01). The same trend continued when we examined the difference between baseline BDNF values and those at 7–10 days after surgery in patients who did not undergo physical prehabilitation (*p* ≤ 0.01). However, it should be noted that BDNF concentrations were significantly higher in PhT patients after termination of the PhT course in comparison to patients who did not undergo prehabilitation (*p* ≤ 0.01) (Figure 3C).

### Binary Logistic Regression Modeling

The following factors influencing the development of POCD in the early postoperative CABG period were recognized: high-level education, the use of preoperative physical rehabilitation, and serum concentrations of S100β and BDNF (Table 4).

**TABLE 4:** Binary regression model values for the prediction of early postoperative cognitive dysfunction (POCD) in patients after CABG.

Variable	B	SE	Wald	Significance	Exp(B)
High-level education (X1)	−0.902	0.442	7.173	0.041	2.464
PhT group (X2)	−0.822	0.419	3.851	0.049	0.44
S100β (X3)	0.056	0.024	5.674	0.017	1.058
BDNF (X4)	−0.00013	0.00005	6.653	0.010	1

*PhT, physical training; S100β, S-100 calcium-binding protein B; and BDNF, brain-derived neurotrophic factor.*

The model sensitivity was 82.1%, specificity was 64%, and the area under the ROC curve was 0.82. A high-level education reduced the risk of early POCD developing, as well as using physical prehabilitation. A higher serum concentration of S100 β and a lower preoperative BDNF value increased the probability of developing of early POCD.

## Discussion

The present study has shown that aerobic PhT during the preoperative period of CABG reduces the incidence of POCD in the early postoperative period. We also established that the integral indicator of attention and overall cognitive status improved after a short course of preoperative training. According to EEG data, patients undergoing physical prehabilitation show a less pronounced postoperative increase in slow-wave theta activity, which may indicate a lower degree of intraoperative brain injury (Tarasova et al., 2013).

Several studies have postulated that PhT can enhance general health and well-being (Aarsland et al., 2010; Sawatzky et al., 2014; Argunova et al., 2017; Doyle et al., 2019). Moreover, PhT is a readily available rehabilitation method for cardiac patients (Argunova et al., 2018; Doyle et al., 2019). Physical rehabilitation has been shown to reduce muscle mass loss and the number of postoperative respiratory complications during the postoperative period of cardiac surgery (Borghi-Silva et al., 2005; Balady et al., 2011; Kamarajah et al., 2020). The study by Argunova et al. (2016) showed that the use of PhT postoperatively reduces cognitive impairment in patients after CABG. However, the effectiveness of physical rehabilitation in the preoperative period of cardiac surgery is currently under active study (Argunova et al., 2017, 2018; Waite et al., 2017). A single-center, randomized controlled trial (PREHAB) showed an increase in physical activity and reduced hospitalization time after preoperative rehabilitation of a 3-week home-based physical exercise program in elderly cancer surgery patients (Waite et al., 2017). However, this study did not assess the functional brain activity, cognitive, and biomarker status and consisted only of cancer surgery patients with senile asthenia who underwent low-intensity physical exercises. Our study demonstrated the beneficial effects of preoperative PhT on cognition in a separate, closely supervised clinical cohort of cardiac surgery patients.

The impact of physical activity on functional organization and compensatory rearrangements of electrical brain activity has been previously shown in a study of traumatic brain injury recovery (Zhavoronkova et al., 2020). The authors demonstrated that combined training (physical activity and cognitive load) was accompanied by hyperreactive EEG rearrangements, especially high-frequency (alpha-beta) bands, with broader inclusion of cortical areas in patients with traumatic brain injury as compared to healthy individuals. It was suggested that the increased activation of local neural networks is evidence of the involvement of compensatory brain resources. We showed that patients with preoperative PhT at 7–10 days after CABG had a lower increase in slow-wave theta activity associated with perioperative ischemic brain damage (Tarasova et al., 2013). The increase of EEG theta power has been associated with mild cognitive impairment and dementia in relation to disease progression (Babiloni et al., 2021). The beneficial effects of PhT on the brain can manifest as increased resistance to acute, global ischemia episodes during on-pump CABG, and electrical wave activity may be indicative of this.

It should also be mentioned that our results suggest the usefulness of serum BDNF, S100β protein, and NSE as biomarkers of the effectiveness of rehabilitation programs in CABG patients. We found that a short course of aerobic exercise was associated with increased serum BDNF concentrations just before surgery. This may indicate a functional role for this neurotrophic factor in training-induced cognitive enhancement in CABG patients. However, BDNF changes may not be the only potential factor that drives improvements in cognitive performance after aerobic exercise. Additionally, patients who underwent a short course of physical prehabilitation had lower serum concentrations of S100β and NSE in the first 24 h after CABG as compared to patients who did not undergo physical prehabilitation. These differences may indicate a lower degree of brain damage in PhT patients. This was also confirmed by the regression analysis results. The regression model indicated that the associations between serum concentrations of S100β and POCD are positive, while those between BDNF and POCD are negative.

We established that NSE, S100, and S100β are sensitive for detecting brain structural and functional damages in patients undergoing various cardiac operations, and that levels peaked at the end of CPB (Yuan and Lin, 2019). Silva et al. (2016) showed that S100β protein is more strongly correlated with the development of cognitive decline after surgery. Thus, it can be assumed that perioperative factors (CPB, anesthesia, and systemic inflammatory response, etc.) lead to blood-brain barrier damage and an increase in neurovascular unit biomarkers. In contrast, preoperative PhT may trigger the expression of neurotrophins.

Our results suggest that using a short course of aerobic PhT during the preoperative period enhances the prevention of early POCD. We propose that the changes in neurovascular unit biomarkers demonstrate protective effects on the brains of patients undergoing a course of physical prehabilitation. These effects are manifested, first, as less pronounced ischemic changes in the brain according to EEG data, and second, as lower incidences of early POCD after on-pump CABG. However, these issues require further study.

## Conclusion

In conclusion, patients undergoing a short preoperative course of PhT have better brain functioning, including indicators of electrical cortical activity, integral cognitive status, and biomarkers of neurovascular unit in the early postoperative CABG period compared to patients who do not undergo physical prehabilitation. We suggest that a short preoperative course of PhT can increase the resistance of the brain to intraoperative damage and reduce cognitive impairment in cardiac patients. Theta wave brain activity and BDNF can be informative markers of the effectiveness of rehabilitation programs.

## Limitations

This study has the limitation that it only used a specific category of male CABG patients. Another limitation of the study is a small set of patients. These issues require that future research on this topic be conducted.

## Data Availability Statement

The datasets presented in this article are not readily available because data sharing is not applicable. Requests to access the datasets should be directed to OB, olb61@mail.ru.

## Ethics Statement

The studies involving human participants were reviewed and approved by the Ethics Committee of Federal State Budgetary Institution of Research Institute of Complex Issues of Cardiovascular Diseases. The patients/participants provided their written informed consent to participate in this study.

## Author Contributions

OT and IT: study concept and design, analysis and interpretation of data, and statistical analysis. EM, DK, YA, SP, and OG: acquisition of data. OB: critical revision of the manuscript and study supervision. IT: drafting of the manuscript. All authors contributed to the article and approved the submitted version.

## Conflict of Interest

The authors declare that the research was conducted in the absence of any commercial or financial relationships that could be construed as a potential conflict of interest.

## Publisher’s Note

All claims expressed in this article are solely those of the authors and do not necessarily represent those of their affiliated organizations, or those of the publisher, the editors and the reviewers. Any product that may be evaluated in this article, or claim that may be made by its manufacturer, is not guaranteed or endorsed by the publisher.
